# Supplementary material for: Palmitoyltransferase DHHC9 and acyl protein thioesterase APT1 modulate renal fibrosis through regulating β-catenin palmitoylation
Source: Nat Commun. 2023 Oct 21;14:6682. doi: 10.1038/s41467-023-42476-z (PMC10590414; doi:10.1038/s41467-023-42476-z)
Supplement: Supplementary file 1 — Supplementary Information [file 41467_2023_42476_MOESM1_ESM.pdf]

Supplementary Materials

Supplementary Figures

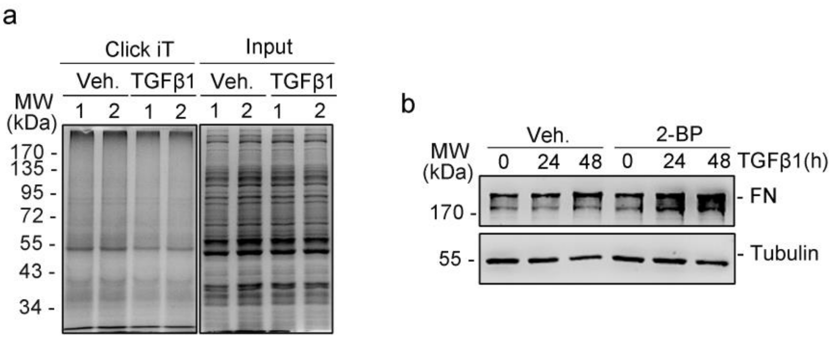

Supplementary Fig. 1. Decreased protein palmitoylation promotes extracellular matrix production in PTCs. a. Click-iT assay showing the decreased protein palmitoylation in PTCs treated with TGFβ1. **b.** Western blot assay showing the induction of FN in PTCs treated with TGFβ1 plus 2-BP. Representative results were obtained from at least three independent experiments with similar results.

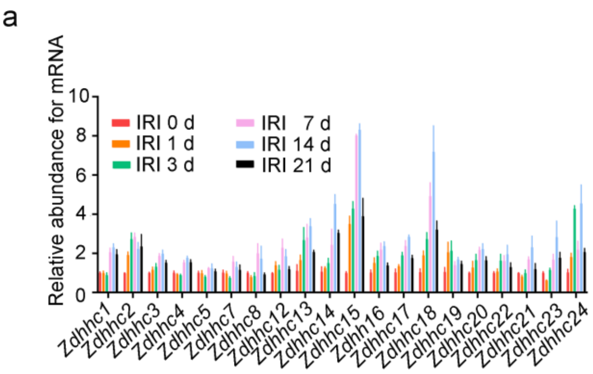

Supplementary Fig. 2. *Zdhhcs* gene expression in kidneys after IRI. a. Quantitative real-time PCR analysis showing the mRNA abundance of *Zdhhc1*-*Zdhhc24* (*Zdhhc6*, *9*, *10*, *11* not included) in mouse kidneys after IRI. *n*=3 biologically independent animals.

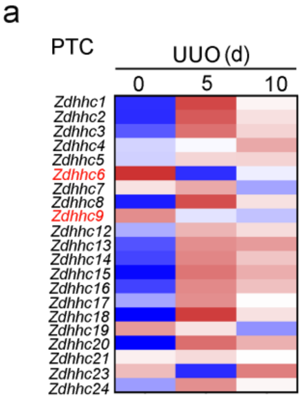

Supplementary Fig. 3. *Zdhhcs* gene expression in tubule from mouse kidneys after UUO. a. Heat map of *Zdhhcs* gene expression in tubule from mice after UUO (From GSE125015).

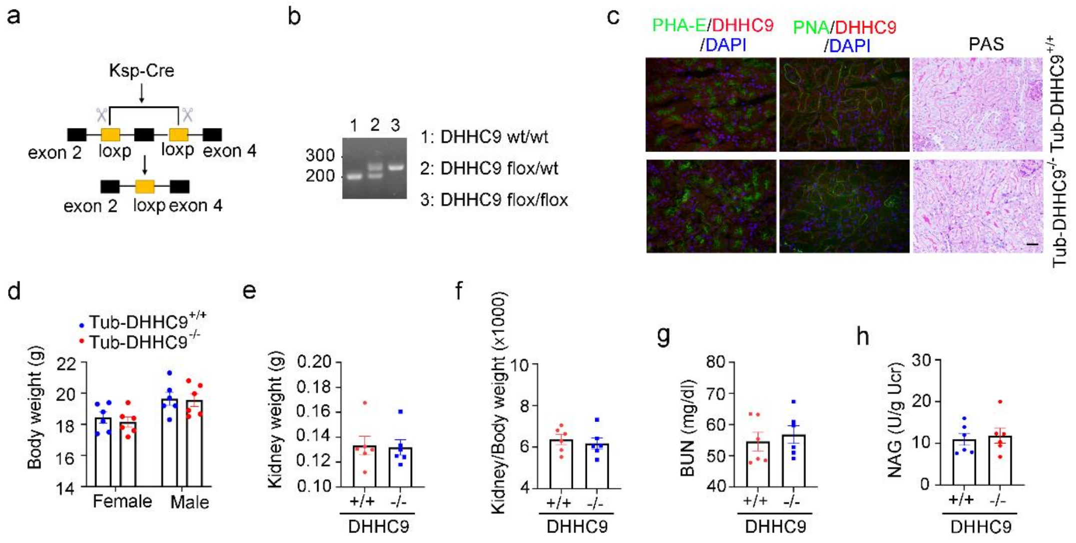

Supplementary Fig. 4. Generating mouse model with tubular cell-specific deletion of DHHC9. a. Strategy for generating mice with tubular cell-specific deletion of DHHC9. **b.** Genotyping the mice with PCR analysis of genomic DNA as indicated. **c.** Costaining for DHHC9 and tubular segment-specific markers, as well as PAS staining in kidneys. Scale bar, 20 μm. **d-h.** The graphs showing the body weight (**d**), kidney weight **(e)**, kidney/body weight index **(f)**, NAG **(g)**, and BUN (**h)** between the knockouts and control littermates at 2 months after birth. *n*=6 biologically independent animals. Representative results were obtained from at least three independent experiments with similar results.

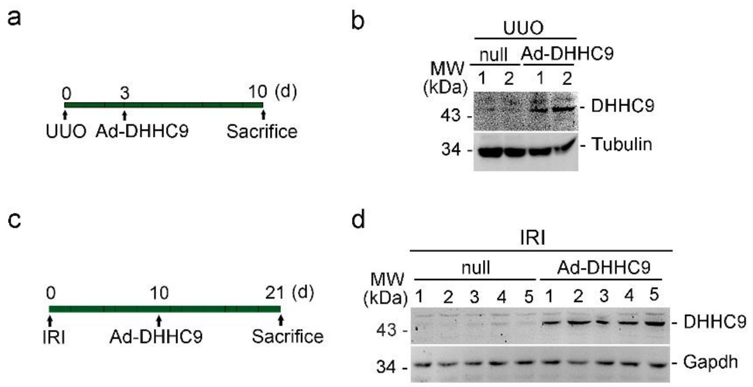

Supplementary Fig. 5. Ad-DHHC9 injection in mouse kidneys. a. Strategy for UUO surgery and Ad-DHHC9 administration in mice. **b.** Western blot analyses showing DHHC9 overexpression in kidneys with Ad-DHHC9 administration. **c.** Strategy for IRI surgery and Ad-DHHC9 administration in mice. **d.** Western blot analyses showing DHHC9 overexpression in kidneys with Ad-DHHC9 administration. Representative results were obtained from at least three independent experiments with similar results.

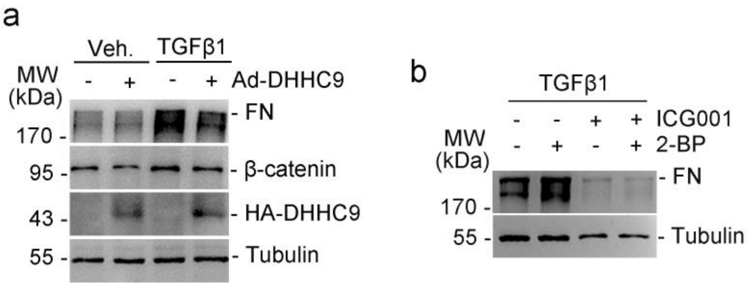

Supplementary Fig. 6. DHHC9 decreases β-catenin abundance and inhibits extracellular matrix production. a. Western blot analyses showing the abundance of FN and β-catenin in PTCs infected with Ad-DHHC9. **b.** Western blot analyses showing FN abundance in PTCs as indicated. Representative results were obtained from at least three independent experiments with similar results.

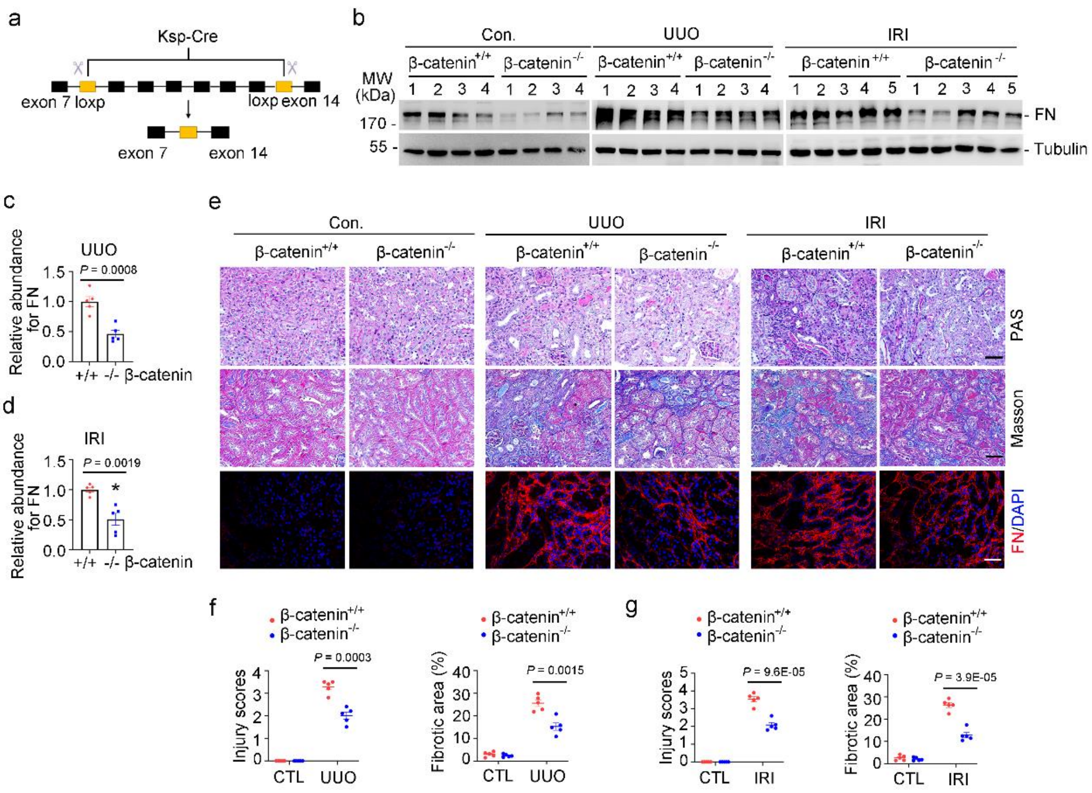

Supplementary Fig. 7. Tubular cell-specific ablation of β-catenin protects against UUO or IRI-induced kidney fibrosis. a. Strategy for generating mice with tubular cell-specific deletion of β-catenin. **b-d.** Western blot assay (**b**) and quantitative analyses (**c, d**) showing ablation of tubular cell β-catenin reduced FN abundance in kidneys after UUO or IRI. *n*=5 biologically independent animals. **e-g.** Representative images of PAS, Masson-trichrome and FN staining in kidneys after UUO or IRI surgery. Scale bar, 20 μm **(e)**, injury scores and the fibrotic area in mice after UUO (**f**) or IRI (**g**). *n*=5 biologically independent animals. Representative results were obtained from at least three independent experiments with similar results. Data represent the mean ± SEM. *P* values were determined by the two-tailed Student's t test.

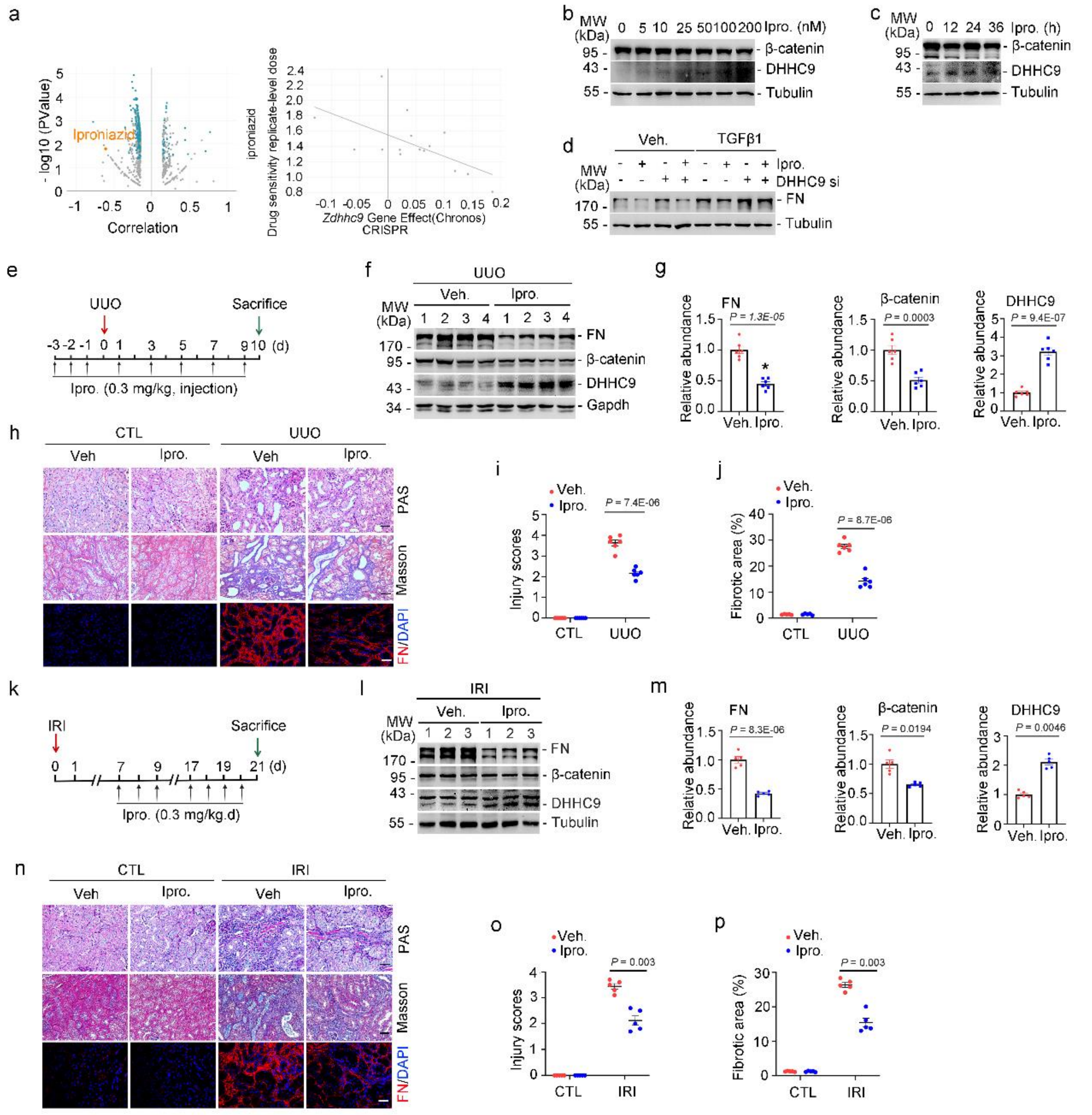

Supplementary Fig. 8. Iproniazid attenuates kidney fibrosis through upregulating DHHC9 expression. a. Identifying iproniazid by screening for drugs with opposite effect to *Zdhhc9* knock out on cell survival (Across the Cancer Dependency Map). **b, c.** Western blot analyses showing the abundance of β-catenin and DHHC9 in PTCs treated with iproniazid at different doses (**b**) or time (**c**). **d.** Western blot analyses showing the effect of iproniazid on FN production in PTCs with or without DHHC9 siRNA transfection. **e.** Strategy for UUO surgery and iproniazid administration in mice. **f, g.** Western blot analyses (**f**) and quantitative analyses (**g**) showing the abundance of FN, DHHC9 and β-catenin in kidneys among groups as indicated. *n*=6 biologically independent

animals. **h-j.** Representative images of PAS, Masson-trichrome and FN staining in kidneys. Scale bar, 20 µm (**h**), injury scores (**i)** and the fibrotic area (**j)**. *n*=6 biologically independent animals. **k.** Strategy for IRI surgery and iproniazid administration in mice. **l, m.** Western blot assay (**l**) and quantitative analysis (**m)** showing the abundance of FN, DHHC9 and β-catenin in kidneys among groups. *n*=5 biologically independent animals. **n-p.** Representative immunohistochemical images of PAS, Masson-trichrome and FN staining in kidneys after IRI surgery. Scale bar, 20 µm (**n**), injury scores (**o),** the fibrotic area (**p)** among groups as indicated. *n*=5 biologically independent animals. Representative results were obtained from at least three independent experiments with similar results. Data represent the mean ± SEM. *P* values were determined by the two-tailed Student's t test.

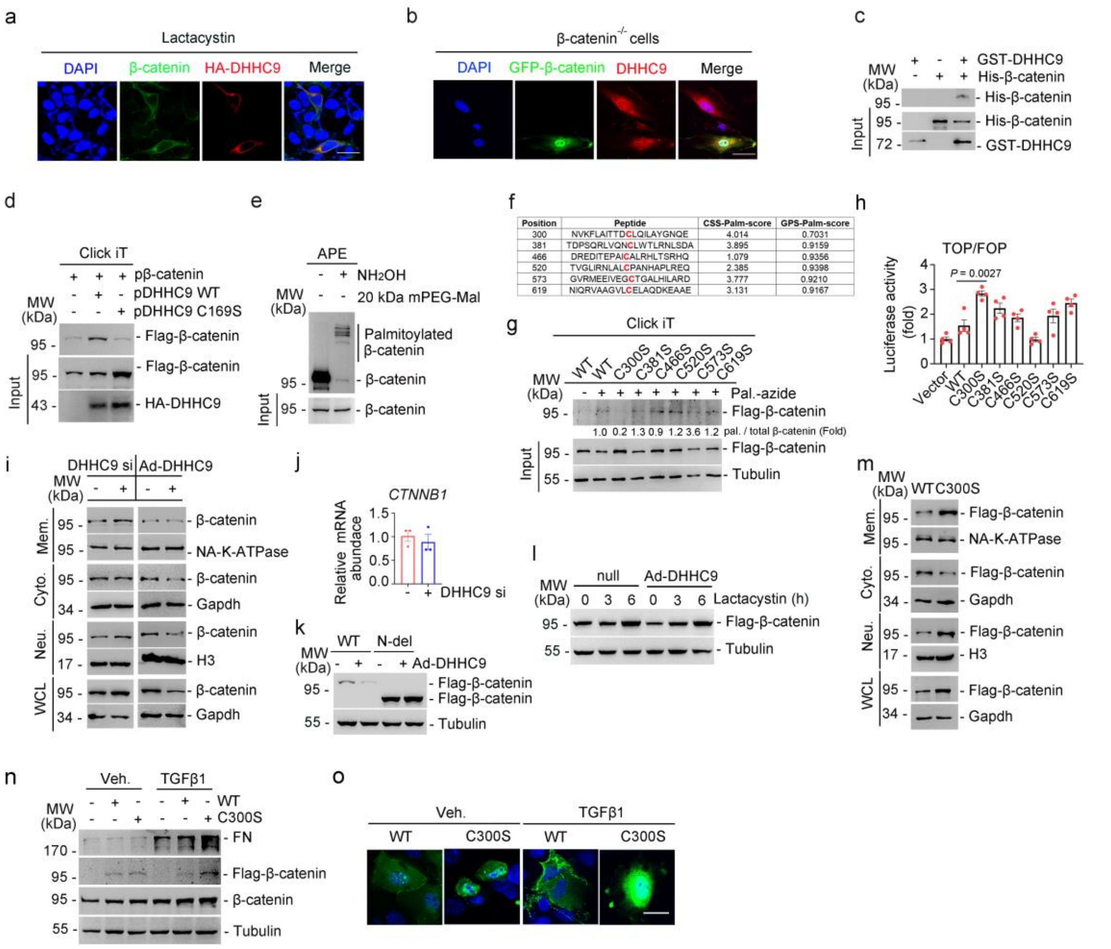

Supplementary Fig. 9. DHHC9 palmitoylates β-catenin at Cys 300 and promotes its degradation. a. Co-staining images showing the co-localization of β-catenin and exogenous DHHC9 in HEK 293A cells treated with lactacystin (20 mM) for 6 hours. Scale bar, 10 μm. **b.** Representative images showing the co-localization of DHHC9 and exogenous GFP-β-catenin in endogenous β-catenin-ablated PTCs. Scale bar, 10 μm. **c.** GST pulldown assay showing the interaction of GST-DHHC9 and His-β-catenin. **d.** Click-iT assay showing the β-catenin palmitoylation. HEK 293A cells were transfected with plasmids containing HA-DHHC9, Flag-β-catenin or HA- DHHC9 C169S mutant as indicated. **e.** APE assays showing the multiple palmitoylation sites in β-catenin molecule. **f.** The predicted palmitoylation sites of β-catenin (From CSS-Palm 4.0 and GPS-Palm software programs). **g.** Click-iT assay and semi-quantitative analyses showing β-catenin palmitoylation status in HEK 293A cells transfected with Flag-β-

catenin or its mutants as indicated. **h.** TOP/FOP-flash luciferase activity in PTCs with β-catenin plasmids transfection. *n*=4 biologically independent samples. **i.** Western blot assay showing the β-catenin distribution in subcellular fractionation in PTCs with DHHC9 siRNA or Ad-DHHC9 transfection. **j.** Quantitative real-time PCR analyses showing the mRNA abundance of β-catenin in PTCs with DHHC9 siRNA transfection. *n*=3 biologically independent samples, *p<0.05. **k.** Western blot assay showing the abundance of N terminal deleted β-catenin in PTCs with Ad-DHHC9 transfection. **l.** Western blot assay showing the abundance of Flag-β-catenin in DHHC9-overexpressed PTCs treated with lactacystin (20 mM). **m.** Western blot assay showing the β-catenin distribution in subcellular fractionation in PTCs transfected with plasmids containing β-catenin or its C300S mutant. **n.** Western blot analyses showing FN abundance in TGFβ1-treated PTCs transfected with plasmids containing β-catenin or its C300S mutant. **o.** Representative fluorescence images showing β-catenin abundance and nuclear translocation in TGFβ1-treated PTCs transfected with plasmids containing β-catenin or its C300S mutant. Scale bar, 10 µm. Representative results were obtained from at least three independent experiments with similar results. Data represent the mean ± SEM. *P* values were determined by the two-tailed Student's t test.

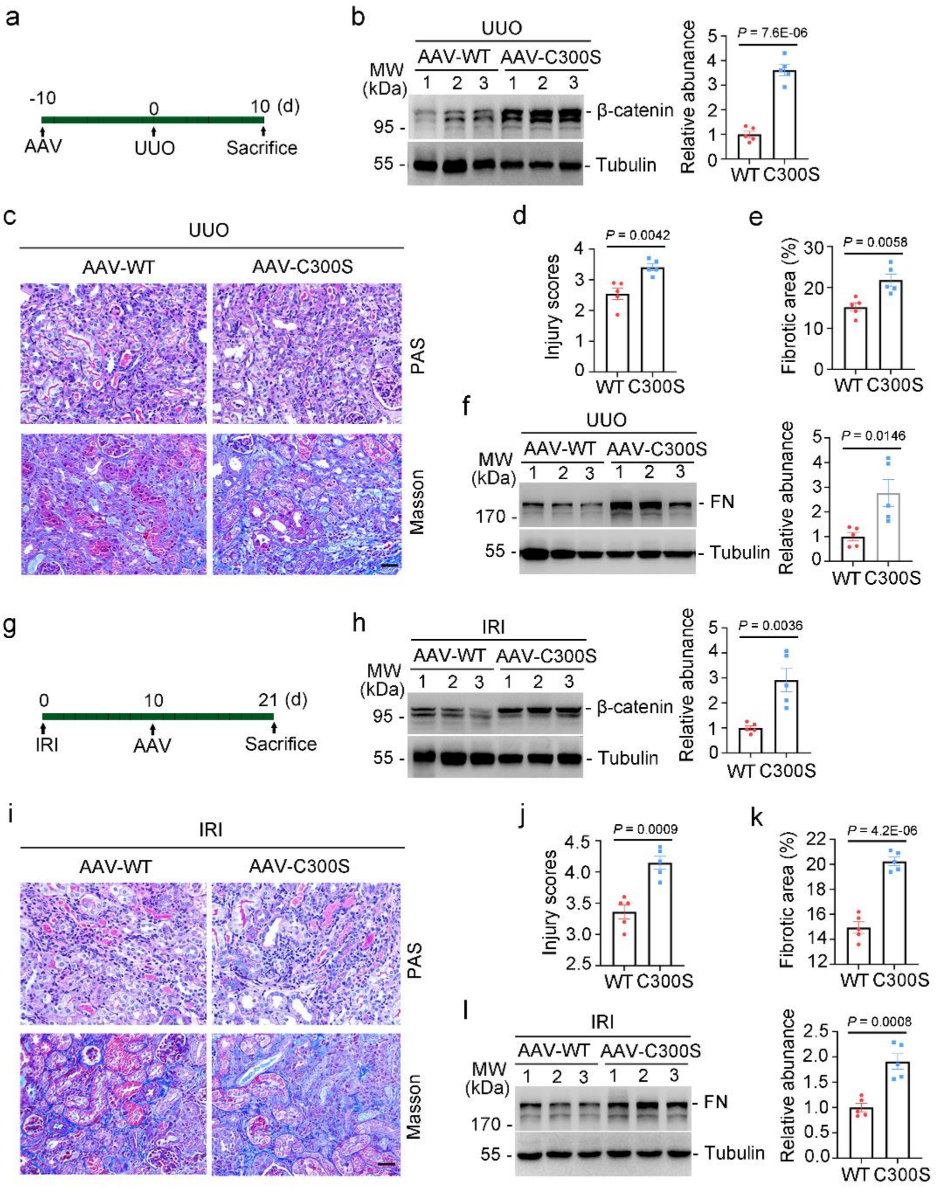

Supplementary Fig. 10. Expression of β-catenin C300S mutant exacerbates renal fibrosis. a. Strategy for UUO surgery and adeno-associated virus (AAV) containing β-catenin administration in mice. AAV-β-catenin WT and AAV-β-catenin C300S were delivered into the kidneys by intraparenchymal injection at day 10 before UUO surgery. **b.** Western blot and quantitative analyses showing β-catenin abundance in kidneys with AAV administration after UUO surgery. **c-e**. Representative images of PAS and Masson-trichrome in kidneys. Scale bar, 20 μm **(c)**, injury scores **(d)** and the fibrotic area **(e)**. *n*=5 biologically independent animals. **f.** Western blot assay and quantitative analysis showing the abundance of FN in kidneys among groups. *n*=5 biologically independent

animals. **g.** Strategy for IRI surgery and adeno-associated virus (AAV) containing β-catenin administration in mice. AAV-β-catenin WT and AAV-β-catenin C300S were delivered into the kidneys by intraparenchymal injection at day 10 after IRI surgery. **h.** Western blot assay and quantitative analyses showing β-catenin expression in kidneys with AAV administration after IRI surgery. **i-k**. Representative images of PAS and Masson-trichrome in kidneys. Scale bar, 20 µm **(i)**, injury scores **(j)** and the fibrotic area **(k)**. *n*=5 biologically independent animals. **l.** Western blot assay and quantitative analysis showing the abundance of FN in kidneys among groups. *n*=5 biologically independent animals. Representative results were obtained from at least three independent experiments with similar results. Data represent the mean ± SEM. *P* values were determined by the two-tailed Student's t test.

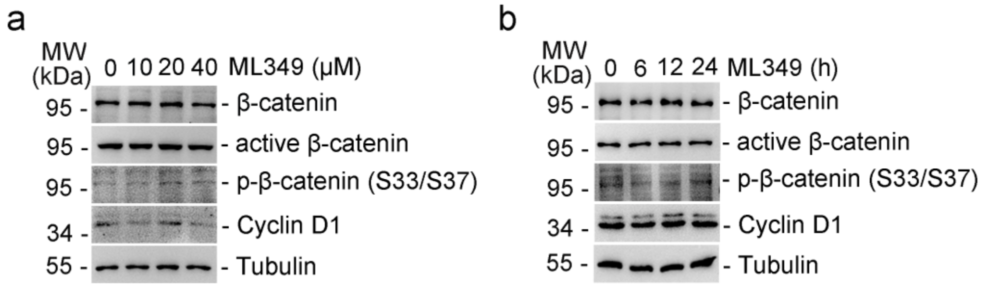

Supplementary Fig. 11. ML349 does not influence β-catenin abundance. a, b. Western blot analyses showing the abundance of β-catenin, its active form, phosphorylated status, and Cyclin D1 in PTCs treated with ML349 at different doses (**a**) or time **(b)**. Representative results were obtained from at least three independent experiments with similar results.

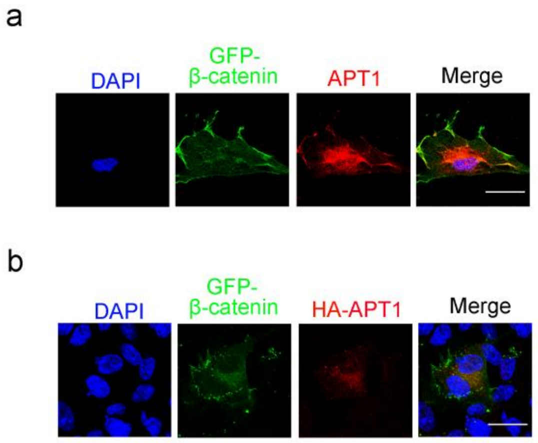

Supplementary Fig. 12. APT1 interacts with β-catenin in HKE 293A cells. a, b. Representative immunofluorescent staining images showing GFP-tagged-β-catenin co-localized with endogenous (**a**) or exogenous APT1 (**b)** in HEK 293A cells. Scale bar, 10 μm. Representative results were obtained from at least three independent experiments with similar results.

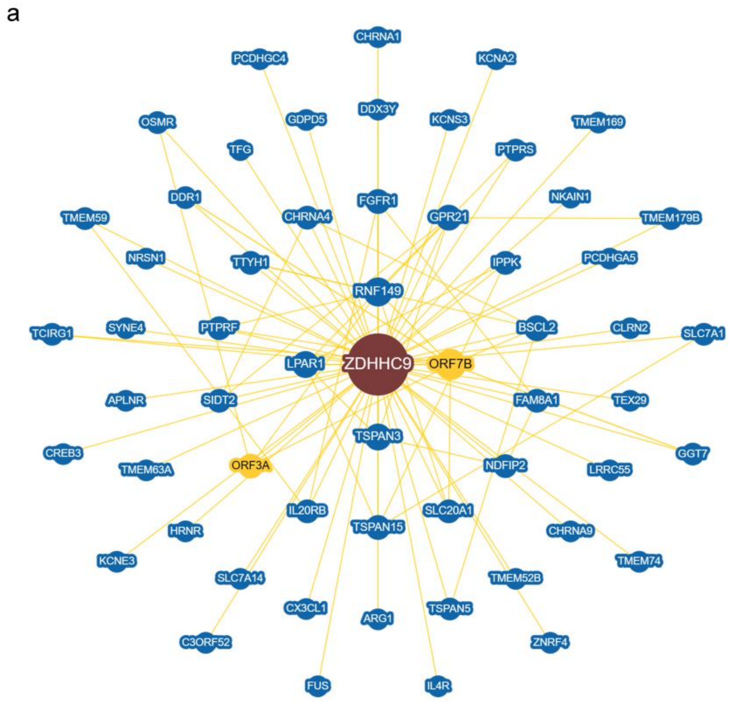

Supplementary Fig. 13. Network diagram of ZDHHC9-interacting proteins. a. Network diagram of ZDHHC9-interacting proteins data from https://thebiogrid.org/119302.

a

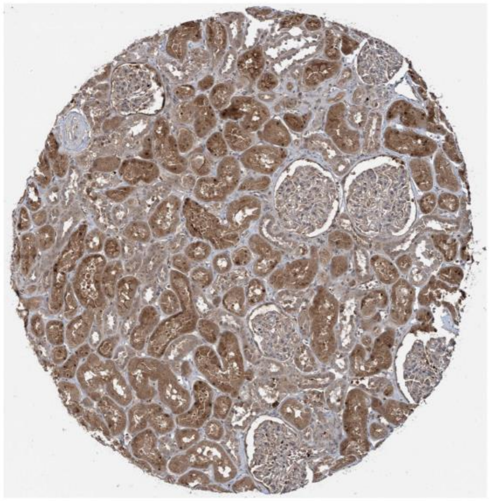

Supplementary Fig. 14. Immumohistochemical staining for DHHC9 in normal human kidney. a. Immumohistochemical staining for DHHC9 in normal human kidney tissue (Male, age 70, Patient id:3356). Data from The Human Protein Atlas.

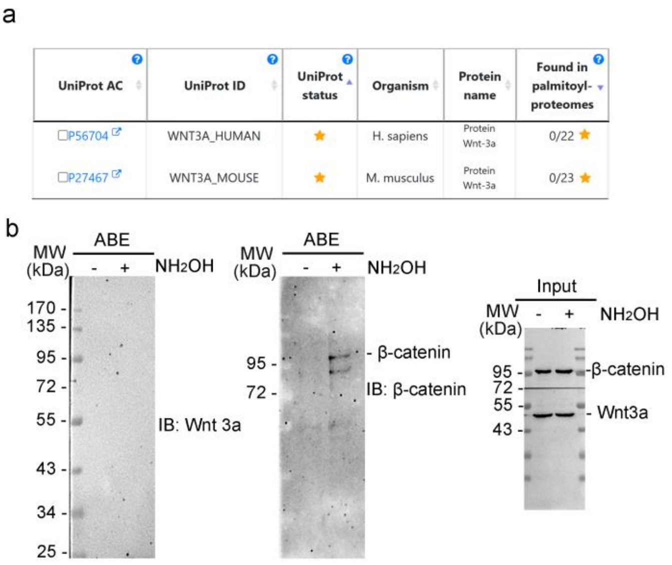

UniProt AC	UniProt ID	UniProt status	Organism	Protein name	Found in palmitoyl-proteomes
P56704	WNT3A_HUMAN	★	H. sapiens	Protein Wnt-3a	0/22 ★
P27467	WNT3A_MOUSE	★	M. musculus	Protein Wnt-3a	0/23 ★

Supplementary Fig. 15. Wnt3a does not undergo S-palmitoylation. a. Wnt3a was not found in protein S-palmitoylation public databases. **b.** ABE assay showing the abundance of S-palmitoylation of Wnt3a or β-catenin. Representative results were obtained from at least three independent experiments with similar results.

Supplementary Table 1. Clinical characteristics of the patients.

CKD patients (*n*=18)						
Number	Age, yrs (Mean± SEM)	Number （Male/Female）	Scr (μM)	BUN (mM)	UPE (g/24h)	Urine occult blood
DN (*n*=6)						
1	54.3±1.8	4/2	116.1	7.31	4.44	+++
2			135.8	9.71	4.67	+++
3			151.7	11.02	1.95	+/-
4			268.5	15.43	1.73	+++
5			128.8	6.02	2.72	+++
6			76	3.86	1.54	+++
IgAN (*n*=6)						
1	44.2±2.8	4/2	172.3	11.7	1.43	+++
2			172.6	10.05	2	+++
3			97.3	4.54	0.2	+++
4			151.4	8.09	1.9	+++
5			86.2	5.82	1.12	+++
6			59.2	3.87	1.387	+++
MN (*n*=6)						
1	45.5±2.7	4/2	115.5	5.51	4.16	+++
2			60.6	4.3	0.28	-
3			70.2	5.95	0.34	-
4			87.8	8.13	5.94	+++
5			130.7	28.54	1.44	++
6			52.2	4.17	3.89	++

Supplementary Table 2. The list of primers used in this study

Genes	Primer Sequence (5'to3')
Mus-Zdhhc1	Sense:ATGAACATCTGCAACAAACCCT
	Antisense:GCTCCATCCATTCCTTCGAGAG
Mus-Zdhhc2	Sense: CTACTACGCCTACGCCATCC
	Antisense: TCCAGCAATTCTTTCTCTGCAT
Mus-Zdhhc3	Sense: ATCCCCACCCATCACTTCC
	Antisense: CTCGGATAAACCACATGGCTC
Mus-Zdhhc4	Sense: TGATTTGTGTTGTCCTGATCTGC
	Antisense: GGAGGCACTGCGGGATTAC
Mus-Zdhhc5	Sense: AAACCCAGCAAGTATGTACCG
	Antisense:AATTGCATTGTAAATGGGCACTG
Mus-Zdhhc6	Sense: ACGTTCACACCATTGCAGAAA
	Antisense: CTTGACTGTGTTCCACCCAAA
Mus-Zdhhc7	Sense: CGGGACATCGAGCACCATC
	Antisense: CGAAGTCTGCATAGACGACGA
Mus-Zdhhc8	Sense: CCCTCTCCTGTGCGCTATG
	Antisense: TCGCCAAAGAGTGAGTCAGTC
Mus-Zdhhc9	Sense: AAGGTGACACGGAAATGGGAG
	Antisense: CGACACTCGAAGGCAAAGAA
Mus-Zdhhc12	Sense: CTCTCCTTCTTCGCGTTAGTG
	Antisense: CTGGCTTTTAGGCACAACAGC
Mus-Zdhhc13	Sense: TCGCAGTGCAGGAATCACAG
	Antisense: GGCAGCCCAGTGAAGAAGA
Mus-Zdhhc14	Sense: CACACTCTCAGACATTATGCCC
	Antisense: TGCATGGTACGGCTATGTGC
Mus-Zdhhc15	Sense: GGTGCCAGTGCTCGTTATTGT
	Antisense: AACTTTTTCCGCTGGACTCAA
Mus-Zdhhc16	Sense: CAGCGAAGTCTGCTGTTGG

	Antisense: CAGCGGATCACATTGTCCAC
Mus-Zdhhc17	Sense: AGACGATAGTTGAACTTGCAGAG
	Antisense: ACCTACACAGTTACCCACCCA
Mus-Zdhhc18	Sense: TCAACGGGCAGACAGTGAAAC
	Antisense: GAAGCGGTAGTTCCGTCTCC
Mus-Zdhhc19	Sense: TGTGACACTTGTGAAGGAACC
	Antisense: AAAAACAGCAGCAGCGTTACA
Mus-Zdhhc20	Sense: GGAAAGACCGTTGTTTACCTTGT
	Antisense: ACTCCTTCTCATAACGCTCCTTC
Mus-Zdhhc21	Sense: ATGGGTCTTCGGATTCACTTTG
	Antisense: CCCTCACTAAGGCAACCAGG
Mus-Zdhhc22	Sense: GGCTGCCTGTTTGTGTGATTG
	Antisense: CCGTGATTCTTTCGCAAGTCTC
Mus-Zdhhc23	Sense: ACTTGCGAAAGAATCACGGAT
	Antisense: ATGGGAAGGGAGGTCAGAACC
Mus-Zdhhc24	Sense: GGATGCCACTGGTACTCACC
	Antisense: CCCAGCACCATCACGTAGG
Mus-Gapdh	Sense: CGGCTCTAATCATAGTTGGGTCT
	Antisense:TGTAGACCATGTAGTTGAGGTCA
Mus-Ctnnb1	Sense: ATGGAGCCGGACAGAAAAGC
	Antisense: TGGGAGGTGTCAACATCTTCTT
