# Supplementary material for: Palmitoyltransferase DHHC9 and acyl protein thioesterase APT1 modulate renal fibrosis through regulating β-catenin palmitoylation
Source: Nat Commun. 2023 Oct 21;14:6682. doi: 10.1038/s41467-023-42476-z (PMC10590414; doi:10.1038/s41467-023-42476-z)
Supplement: Supplementary file 3 — Reporting Summary [file 41467_2023_42476_MOESM3_ESM.pdf]

nature portfolio

Corresponding author(s): Chunsun Dai

Last updated by author(s): 2023-10-08

Reporting Summary

Nature Portfolio wishes to improve the reproducibility of the work that we publish. This form provides structure for consistency and transparency in reporting. For further information on Nature Portfolio policies, see our Editorial Policies and the Editorial Policy Checklist.

Statistics

For all statistical analyses, confirm that the following items are present in the figure legend, table legend, main text, or Methods section.

n/a	Confirmed	
☐	☒	The exact sample size (*n*) for each experimental group/condition, given as a discrete number and unit of measurement
☐	☒	A statement on whether measurements were taken from distinct samples or whether the same sample was measured repeatedly
☐	☒	The statistical test(s) used AND whether they are one- or two-sided<br>*Only common tests should be described solely by name; describe more complex techniques in the Methods section.*
☒	☐	A description of all covariates tested
☒	☐	A description of any assumptions or corrections, such as tests of normality and adjustment for multiple comparisons
☐	☒	A full description of the statistical parameters including central tendency (e.g. means) or other basic estimates (e.g. regression coefficient) AND variation (e.g. standard deviation) or associated estimates of uncertainty (e.g. confidence intervals)
☒	☐	For null hypothesis testing, the test statistic (e.g. *F*, *t*, *r*) with confidence intervals, effect sizes, degrees of freedom and *P* value noted<br>*Give P values as exact values whenever suitable.*
☒	☐	For Bayesian analysis, information on the choice of priors and Markov chain Monte Carlo settings
☒	☐	For hierarchical and complex designs, identification of the appropriate level for tests and full reporting of outcomes
☒	☐	Estimates of effect sizes (e.g. Cohen's *d*, Pearson's *r*), indicating how they were calculated

Our web collection on statistics for biologists contains articles on many of the points above.

Software and code

Policy information about availability of computer code

Data collection	The intensity of immunoblotting bands was quantified by the Image J software (v1.53). Fibrotic area is quantified by Image-Pro Plus software(v6.0). Images of protein immunoblots were taken by Tanon-5200Multi
Data analysis	All statistical graphs were performed using the GraphPad Prism software (v 9.0.2).All software used in this study are either commercially available or open source.

For manuscripts utilizing custom algorithms or software that are central to the research but not yet described in published literature, software must be made available to editors and reviewers. We strongly encourage code deposition in a community repository (e.g. GitHub). See the Nature Portfolio guidelines for submitting code & software for further information.

Data

Policy information about availability of data

All manuscripts must include a data availability statement. This statement should provide the following information, where applicable:

- Accession codes, unique identifiers, or web links for publicly available datasets
- A description of any restrictions on data availability
- For clinical datasets or third party data, please ensure that the statement adheres to our policy

The authors declare that all data supporting the findings of this study are available within the article and its Supplementary Information files, or from the corresponding author. Databases used in this study include GEO Datasets (GSE12501,https://www.ncbi.nlm.nih.gov/geo/query/acc.cgi?acc=GSE125015; GSE98622,https://www.ncbi.nlm.nih.gov/geo/query/acc.cgi?acc=GSE98622), Human Protein Atlas (https:// www.proteinatlas.org), Database of Protein, Genetic and [illegible]

Chemical Interactions (https://thebiogrid.org/), Database on Protein S-palmitoylation (https://swisspalm.org/) and Kidney Single Cell Datasets (http://humphreyslab.com/SingleCell/).

Human research participants

Policy information about studies involving human research participants and Sex and Gender in Research.

Reporting on sex and gender	Findings from this study do not apply to only one sex or gender and consent has been obtained for sharing of individual level data.
Population characteristics	Information about the kidney biopsy patient sex, age and kidney function status are given in the source data of Supplementary Table 1.
Recruitment	The samples of renal biopsies were obtained from Center for Kidney Diseases, the Second Affiliated Hospital of Nanjing Medical University. The sample should be representative of the populations served by the hospital and reflect the general pathological characteristics of Diabetic nephropathy (DN), IgA nephropathy (IgA) or Membranous nephropathy (MN)
Ethics oversight	The use of patient specimens and the relevant database were approved by the Institutional Review Board at the Second Affiliated Hospital of Nanjing Medical University.

Note that full information on the approval of the study protocol must also be provided in the manuscript.

Field-specific reporting

Please select the one below that is the best fit for your research. If you are not sure, read the appropriate sections before making your selection.

☒ Life sciences ☐ Behavioural & social sciences ☐ Ecological, evolutionary & environmental sciences

For a reference copy of the document with all sections, see nature.com/documents/nr-reporting-summary-flat.pdf

Life sciences study design

All studies must disclose on these points even when the disclosure is negative.

Sample size	Sample sizes were determined on the basis of the pilot and the published studies. Studies involving animal models with a sample size of at least 5 per group and cellular studies with at least three repetitions
Data exclusions	No data was excluded from this study.
Replication	Results were confirmed in at least three biological replicates for each experiment unless otherwise stated.
Randomization	The samples for each experiment were randomized to be examined.
Blinding	UUO and IRI surgeries were performed by an operator blind to the experimental settings and mouse genotype.

Reporting for specific materials, systems and methods

We require information from authors about some types of materials, experimental systems and methods used in many studies. Here, indicate whether each material, system or method listed is relevant to your study. If you are not sure if a list item applies to your research, read the appropriate section before selecting a response.

Materials & experimental systems

n/a	Involved in the study	
☐	☒	Antibodies
☐	☒	Eukaryotic cell lines
☒	☐	Palaeontology and archaeology
☐	☒	Animals and other organisms
☒	☐	Clinical data
☒	☐	Dual use research of concern

Methods

n/a	Involved in the study	
☒	☐	ChIP-seq
☒	☐	Flow cytometry
☒	☐	MRI-based neuroimaging

March 2021

Antibodies

Antibodies used	Anti-fibronectin antibody produced in rabbit (cat: F3648, Sigma-Aldrich),<br>Mouse monoclonal anti-Tubulin (RRID: AB_630403, cat: sc53646),<br>Anti-ZDHHC9 antibody produced in rabbit (cat: SAB4502104, Sigma-Aldrich),<br>HA-Tag (6E2) Mouse mAb (cat: 2367S, Cell signaling technology),<br>GAPDH polyclonal antibody (cat: AP0063, Bioworld),<br>β-Catenin (6B3) Rabbit mAb (cat: 9582S, Cell signaling technology),<br>Phospho-β-Catenin (Ser33/37) Antibody (cat: 2009S, Cell signaling technology),<br>Non-phospho (Active) β-Catenin (Ser45) (D2U8Y) Rabbit mAb (cat: 19807S, Cell signaling technology),<br>Anti-Cyclin D1 (cat: 2922S, Cell signaling technology),<br>Monoclonal ANTI-FLAG® M2 antibody produced in mouse (cat: F1804, Sigma-Aldrich),<br>Na, K-ATPase α1 (D4Y7E) Rabbit mAb (cat: 23565S, Cell signaling technology),<br>Histone H3 (D1H2) XP® Rabbit mAb (cat: 4499S, Cell signaling technology),<br>Axin1 (C76H11) Rabbit mAb (cat: 2087S, Cell signaling technology),<br>Casein kinase I alpha antibody (H-7) (cat: sc-74582, Santa Cruz Biotechnology),<br>GSK-3β (27C10) Rabbit mAb (cat: 9315S, Cell signaling technology),<br>Anti-Ubiquitin (cat: sc-8017, Santa Cruz),<br>LYPLA1 (K90) polyclonal antibody (cat: BS3063,Bioworld),<br>anti-Normal Mouse IgG (cat:G-21040, Thermo Fisher Scientific)<br>anti-Normal Rabbit IgG (cat:21234,Thermo Fisher Scientific)
Validation	All of the antibodies used were validated either directly by the commercial manufacturers or by cited references included on the antibody information for the specified uses in our manuscript

Eukaryotic cell lines

Policy information about cell lines and Sex and Gender in Research

Cell line source(s)	Primary tubular epithelial cells were isolated from the kidneys of 2-week-old newborn mice. Both males and females were used because the mice were too young in age for sex differentiation
Authentication	Primary tubular epithelial cells extracted by this method have been confirmed in our prior research (PMID:35483524) by cytokeratin18 (epithelial cell maker) staining
Mycoplasma contamination	All cell lines used in this study were negative for the tests of mycoplasma contamination.
Commonly misidentified lines<br>(See ICLAC register)	No commonly misidentified cell lines were use

Animals and other research organisms

Policy information about studies involving animals; ARRIVE guidelines recommended for reporting animal research, and Sex and Gender in Research

Laboratory animals	The Ksp-Cre transgenic mice were ordered from Jackson Laboratory (cat: 012237; C57BL/6J background). Mice with a floxed DHHC9 allele (DHHC9 floxed mice; exon 3 flanked by loxP sites) and mice with a floxed APT1 allele (APT1 floxed mice; exon 4 to 5 flanked by loxP sites) were ordered from Cyagen (cat: S-CKO-05388 and S-CKO-04319, respectively; C57BL/6J background). Mice with a floxed β-catenin allele (β-catenin floxed mice; exon 8 to 13 flanked by loxP sites) were ordered from GemPharmatech (cat: T052262; C57BL/6J background). 6-8 weeks old Zdhhc9, APT1, or β-catenin floxed mice were crossed with 6-8 weeks old Ksp-Cre mice respectively to generate offsprings with tubular cell deletion of DHHC9 (Tub-DHHC9 -/-, genotype: Cre+/-, DHHC9fl/fl), APT1 (Tub-APT1-/-, genotype: Cre+/-, APT1fl/fl), or β-catenin (Tub-β-catenin-/-, genotype: Cre+/-, β-cateninfl/fl), respectively. The same gender with genotyping Cre-/-, from the same litters were considered as control littermates. Taking DHHC9 deletion model as an example, Tub-DHHC9-/- and control littermates, aged between 8 and 10 weeks, were subjected to UUO or IRI operation to induce kidney fibrosis. Mice were housed in a pathogen-free environment with the temperature maintained at 23 ± 2°C and relative humidity at 50 to 65% under a 12 h/12 h light/dark cycle with free access to food and water.
Wild animals	No wild animals were used.
Reporting on sex	The animal model used in this study was male mice. Previous studies have reported that female mice are less sensitive to ischemia-reperfusion injury than male mice(PMID: 34853151). In order to ensure the successful construction of the IRI model and reduce the error caused by gender differences, we used male mice in both models of renal fibrosis
Field-collected samples	No field-collected samples were used.
Ethics oversight	All animals were maintained in Specific Pathogen-Free Laboratory Animal Center of Nanjing Medical University, according to the guidelines of the Institutional Animal Care and Use Committee from Nanjing Medical University (IACUC-2112054).

Note that full information on the approval of the study protocol must also be provided in the manuscript.
